# Night shift work before and during pregnancy in relation to depression and anxiety in adolescent and young adult offspring

**DOI:** 10.1007/s10654-019-00525-2

**Published:** 2019-05-13

**Authors:** Susanne Strohmaier, Elizabeth E. Devore, Celine Vetter, A. Heather Eliassen, Bernard Rosner, Olivia I. Okereke, Eva S. Schernhammer

**Affiliations:** 1 Channing Division of Network Medicine, Brigham and Women’s Hospital and Harvard Medical School, Boston, MA USA; 2 Department of Epidemiology, Center for Public Health, Medical University of Vienna, Kinderspitalgasse 15, 1090 Vienna, Austria; 3 Department of Integrative Physiology, University of Colorado, Boulder, CO USA; 4 Department of Epidemiology, Harvard T.H. Chan School of Public Health, Boston, MA USA; 5 Department of Biostatistics, Harvard T.H. Chan School of Public Health, Boston, MA USA; 6 Department of Psychiatry, Massachusetts General Hospital, and Harvard Medical School, Boston, MA USA

**Keywords:** Circadian disruption, Intergenerational, Night shift work, Offspring mental health

## Abstract

We investigated the relationship between maternal history of nightshift work before and shift work during pregnancy and offspring risk of depression and anxiety, among mothers participating in the Nurses Health Study II and in their offspring enrolled in the Growing Up Today Study 2 between 2004 and 2013. Case definitions were based on offspring self-reports of physician/clinician-diagnosed depression and/or anxiety, regular antidepressant use and depressive symptoms assessed using the Center for Epidemiologic Studies Depression Scale. Multivariable-adjusted odds ratios (ORs) and 95% confidence intervals (CIs) were estimated using generalized estimating equation models. We found no associations between maternal nightshift work before pregnancy or during pregnancy and offspring mental health disorders (e.g., nightshift work before pregnancy: depression (based on physician/clinician diagnosis): OR_ever nightwork _= 1.14; 95% CI, 0.88–1.47; either depression or anxiety: OR_ever nightwork _= 0.93; 95% CI, 0.81–1.08; nightshift work during pregnancy: depression: OR_ever nightwork _= 1.14; 95% CI, 0.68–1.94; depression or anxiety: OR_ever nightwork _=1.17; 95% CI, 0.70–1.98) and no dose-response relationship with longer history of nightshift work (all P_Trend  _>0.10). Stratifying by maternal chronotype revealed a higher risk of depression for offspring whose mothers worked nightshifts before pregnancy and reported being definite morning chronotypes (a proxy for circadian strain) (OR_ever nightwork _= 1.95; 95% CI, 1.17, 3.24 vs. OR_ever nightwork _= 0.93; 95% CI, 0.68, 1.28 for any other chronotype; P_Interaction _= 0.03). Further studies replicating our findings and refined understanding regarding the interplay of nightshift work and chronotype and its potential influences on offspring mental health are needed.

**Electronic supplementary material:**

The online version of this article (10.1007/s10654-019-00525-2) contains supplementary material, which is available to authorized users.

## Introduction

In the United States and worldwide, depression and anxiety are leading causes of morbidity and disability in young people; both negatively impact long-term health and pose a serious public health problem [1–3]. In 2015, the National Institute of Mental Health [4, 5] reported that 12.5% of the U.S. population aged 12 to 17 had experienced at least one major depressive episode in the past year and 10.3% of the population aged 18 to 25. Further, a twelve-month prevalence of any mental illness (including depression and anxiety) of 21.7% was reported in that age group, following diagnostic criteria stated in the Diagnostic and Statistical Manual of Mental Disorder (DSM-IV) [6]. While prevalence estimates vary by country possibly due to different screening scales, clinical cut-points, or application of diagnostic criteria, the age-at-onset for most mental disorders is consistently reported with a median in the early- to mid-twenties across countries [7].

Established risk factors for depression and anxiety include individual-level characteristics (e.g., sex, age, psychosocial stress, substance use) and family-level characteristics (e.g., parental education, socio-economic status, family structure, family history) [8]. However, there is growing evidence that psychiatric disorders may have developmental origins, and maternal exposure may impact offspring mental health via fetal programming [9, 10].

Work schedules involving rotating night shift work have previously been linked to mental disorders [11–15] and other chronic diseases such as type 2 diabetes [16], cardiovascular diseases [17] and several cancers [18, 19], with some evidence that inter-individual differences in chronotype could modify these relationships [19, 20]. Moreover, recent studies suggest that shift working nurses experience epigenetic alterations (e.g., methylation patterns in the promoter region of the serotonin transporter gene) [21, 22], which could contribute to development of depressed mood in offspring [23].

Additionally, night shift work surrounding pregnancy, through regulation of the maternal melatonin profile, may lead to behavioral programming in the offspring via alterations of the hypothalamic-pituitary-adrenal (HPA) axis [24, 25]. More specifically, melatonin can freely cross the placenta and provide photoperiodic information to the fetus [26] and in turn influence the development of the circadian system [27]. Evidence form animal models shows that maternal melatonin is involved in the development of the fetal adrenal gland and glucocorticoid signaling [28] which are reportedly important in the programming of emotional behaviors [29].

Animal models also revealed that parental social defeat stress prior to conception can lead to depression and anxiety behaviors in offspring rodents [30]. In humans, so far only extreme stressors prior to conception or during pregnancy (e.g., Holocaust, World Trade Center attack) have been linked to adverse mental outcomes in offspring [31–33].

However, no prior study has examined the association of night shift work (an established health stressor [34]) before and during pregnancy, maternal differences in preferred wake/sleep time (i.e., chronotype), and offspring mental health. To provide insights regarding these associations, we combined existing shift work information in > 4000 women participating in the Nurses’ Health Study II with information on lifetime risk of depression and anxiety in their offspring, who were enrolled in the Growing Up Today Study 2.

## Methods

### Study population

Our present analyses included mother–child pairs, comprised of mothers who were participants in the prospective Nurses’ Health Study II (NHSII) and their children who were participants in the Growing Up Today Study 2 (GUTS2). NHSII is an ongoing prospective cohort study. It was established in 1989, when 116,429 US female nurses aged 25–42 years responded to a mailed questionnaire about their health and lifestyle; biennial follow-up questionnaires are used to update information on risk factors and medical history.

GUTS2 was established in 2004, when after obtaining maternal consent, invitation letters and questionnaires were sent to 17,280 children aged 9 to 15 years, born to NHSII participants between 1987 and 1995. Of these, 10,918 children returned completed questionnaires and information on health and lifestyle factors with updated information from follow-up questionnaires in 2006, 2008, 2011, and 2013.

This study has approval from the Committees on the Use of Human Subjects in Research at the Brigham and Women’s Hospital and the Harvard T.H. Chan School of Public Health (Boston, MA, USA). If participants returned the baseline self-administered questionnaire, it was assumed to imply informed consent in both cohorts.

### Ascertainment of night shift work among mothers

#### Mother’s history of rotating night shift work before pregnancy

Information on the history of rotating night shift work was first obtained in the NHSII baseline questionnaire in 1989, when nurses were asked to report their total years of rotating night shift work, defined as “at least three nights per month in addition to working days or evenings in the respective month”. Using updated information from questionnaires in 1991 and 1993 and retrospective assessments for the period between 1993 and 1995 from the questionnaire in 2001, cumulative shift work exposure was derived by adding together the number of years participating in shift work before conception, for children born between 1989 and 1995.

#### Mother’s night shift work exposure during pregnancy

Additional information regarding specific occupational exposures, including shift work during the most recent pregnancy since 1993, was collected through a supplemental questionnaire following the main NHSII questionnaire in 2001. Participants who had previously reported at least one pregnancy since 1993, worked as a nurse and indicated their willingness to participate were sent additional questions that asked about working schedules and number of night shifts per month for each trimester, along with other exposures.

### Ascertainment of mental health disorders among offspring

**Fig. 1:**
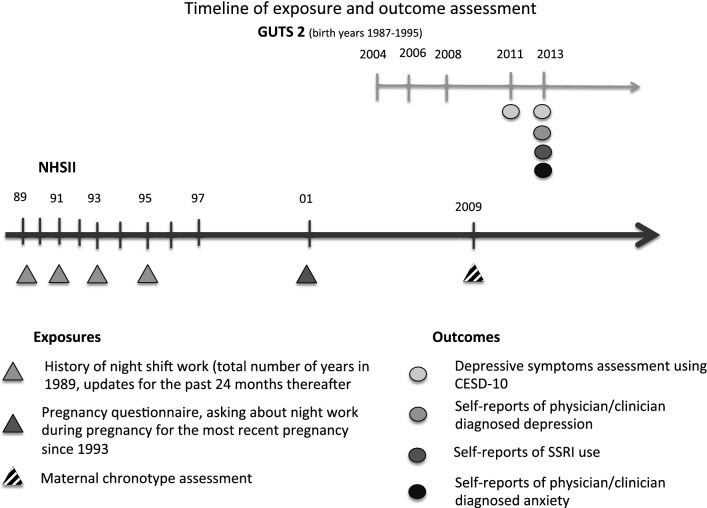
Timeline of exposure assessment in NHSII and outcome assessment in GUTS2

In this study, we utilized multiple sources of information on depression. First, we defined depression cases through self-report of physician/clinician-diagnosed depression, which was collected on the 2013 GUTS questionnaire. At that time, participants were also asked to indicate the year of first diagnosis (before 2006, 2006–2008, 2009–2011, 2012 or later). An alternative, broader Boolean OR case definition was based on reporting a physician/clinician-diagnosed depression, regular antidepressant use, or the presence of clinically significant depressive symptoms [35]. Information on selective serotonin reuptake inhibitors (SSRIs) use was obtained from the 2013 GUTS questionnaire, when participants were asked about regular medication use in the past 12 months. Depressive symptoms were assessed using the Center for Epidemiological Studies Depression Scale-10 (CESD-10) [36, 37] in 2011 and 2013. The presence of clinically significant depressive symptoms was defined as having a CESD-10 score ≥10 in either 2011 or 2013 [38]; the CESD-10 ≥ 10 cutoff has been previously validated for major depression. Cases of anxiety were defined based on self-report of physician/clinician-diagnosed anxiety on the 2013 GUTS questionnaire. A composite psychiatric outcome was created, including participants as cases if they: self-reported diagnosis of depression or anxiety, used antidepressant medication, and/or scored ≥10 points on the CESD-10 at any time. A timeline of exposure and outcome assessments is shown in Fig. 1.

### Ascertainment of covariates

To calculate maternal age at delivery, we computed the difference between mother’s and child’s dates of birth. Maternal diet quality was characterized based on the validated food frequency questionnaire (FFQ) [39] from the 1991 main NHSII questionnaire, from which the Alternative Healthy Eating Index (AHEI) and alcohol intake were derived [40]. In 1989, participants were asked to report frequency of various physical activities, which was used to derive energy expenditure in metabolic equivalent (MET) hours per week [41]. Information on smoking habits and weight and height was collected biennially; this was applied in determining the status of smoking and anthropomorphic measures that were most proximal to and before conception of the first included child. Maternal diagnosis of diabetes and high blood pressure before conception were ascertained on the NHSII baseline questionnaire in 1989, and maternal lifetime history of depression was self-reported in NHSII beginning in 2003. Maternal chronotype was assessed in 2009 using a single question from the Morningness-Eveningness Questionnaire [42], which has been validated for classifying individuals as “definitely a morning type”, “rather more a morning than an evening type”, “rather more an evening than a morning type”, “definitely an evening type” or neither.

To capture the family’s socioeconomic status surrounding a child’s birth and early childhood, we used US Census tract data [43] from the 1989 U.S. Census. We considered the variables median income and percentage of the population holding a college degree in the mother’s neighborhood, which could be extracted based on the mother’s address at that time. Additionally, we ascertained information on husbands’ level of education in 1999, mother’s subjective social and economic standing in her community and the US, and mother’s adulthood household income reported in 2001. Further, we considered information on the geographic region children were living at the GUTS2 baseline in 2004.

The GUTS2 baseline questionnaire also assessed offspring stage of pubertal development (Tanner stage), using a validated scale of pubic hair illustrations [44]. Further, offspring sedentary behavior was derived by summing up the reported time they had spent watching television, using the computer, surfing on the internet, and reading/doing homework.

### Final samples for analyses

Since history of maternal rotating night shift was assessed for the first time in 1989, we only considered children born in 1989 or later in our analyses; hence, we excluded 4721 of the original cohort of 10,918 children. Further, we excluded twins and triplets (195 children, 87 mothers), children born at less than 37 weeks gestation (i.e., non full-term pregnancies) (1183 children, 1001 mothers), and mother-child pairs with missing exposure information (7 children, 6 mothers). In total, we included 4813 children born to 4044 mothers in our analyses of pre-conception shift work and mental health outcomes in offspring.

To assess the associations between night shift work during pregnancy and depression and anxiety in offspring, we identified a subset of 621 GUTS2 participants whose mothers participated in the occupational supplemental questionnaire. After excluding children born at less than 37 weeks gestation (72 children, 72 mothers) and pairs with missing exposure information (4 children, 4 mothers), a total of 545 mother-child pairs were left for these analyses.

### Statistical analysis

#### Mother’s history of rotating night shift exposure before conception

To account for clustering within families, we used generalized estimating equation (GEE) regression models specifying a logit link function and an exchangeable correlation structure to estimate odds ratios (ORs) and 95% confidence intervals (CIs) for depression and anxiety outcomes in offspring across four categories of cumulative rotating night shift work before conception (none, < 3 years, 3–5 years, and **≥ **6 years). Mothers without a history of night shift work were considered the reference group. We also used this approach to examine outcomes in offspring comparing mothers who ever worked night shifts versus mothers who never worked night shifts.

Initial models were adjusted for offspring baseline age and sex (basic model). We further considered a wide range of covariates describing maternal lifestyle and social economic status, including BMI and smoking status before pregnancy, AHEI dietary score, physical activity, husband’s education, geographic region at GUTS baseline, and the Census tract education rate in 1989 in multivariate model 1 (MV model 1). Lastly, maternal lifetime history of depression was added to the covariates included in MV model 1 (MV model 2). We included a missing indicator in our models when covariates had missing values.

#### Mother’s night shift work exposure during pregnancy

Based on the information provided in the supplemental questionnaire, we used logistic regression models to estimate odds ratios and 95% CIs for offspring depression and anxiety comparing children born to mothers who worked night shifts during pregnancy to those whose mothers did not. Covariates were added to the models following a similar selection procedure as described above.

Previous studies have identified associations between chronotype and mental health outcomes [45, 46], and effect modification of associations between night shift work and chronic diseases by chronotype [19, 20]. Therefore, we assessed possible effect modification of the association between night shift work and offspring mental health outcomes by maternal chronotype. To test for effect modification, we included multiplicative interaction terms in multivariable regression models.

All analyses were conducted using SAS version 9.4 (SAS Institute, Cary, NC) and all statistical tests were two-sided and considered statistically significant at *P *< 0.05.

## Results

### Mother’s history of rotating night shift work before pregnancy

The 4044 mothers included in our analyses were, on average, 33.4 (SD 3.6) years old when they delivered their children in this study, while the 4813 children were on average 11.7 (SD 1.2) years old when they started participating in GUTS2. There were more girls than boys included in the study (53.4% girls, 46.6% boys). Comparing maternal and offspring characteristics across categories of duration of night shift work before conception (65% of the mothers had reported night shift work), we found only modest differences (Table 1). Mothers reporting longer durations of night shift work were slightly older at birth, and more likely to be past or current smokers and to adhere to a healthy diet (AHEI); they also reported a slightly higher BMI before pregnancy and a higher frequency of lifetime physician/clinician-diagnosed depression.

**Table 1:** Maternal and offspring characteristics *by rotating night shift work history before pregnancy*, for 4044 mothers of a total of 4813 children, born between 1989 and 1995, enrolled in the Growing Up Today Study 2

	History of rotating night shift work									
Characteristic	Never worked rotating night shifts		< 3 years		3–5 years		≥ 6 years		Ever worked rotating night shifts	
	(n = 1424)		(n = 1254)		(n = 946)		(n = 420)		(n = 2620)	
	Mean (SD)	%^d^	Mean (SD)	%	Mean (SD)	%	Mean (SD)	%	Mean (SD)	%
Maternal age at delivery	33.1 (3.5)		33.2 (3.6)		33.4 (3.5)		35.0 (3.2)		33.6 (3.5)	
BMI before pregnancy^a^	22.7 (3.9)		22.7 (3.8)		23.0 (3.9)		23.3 (4.2)		22.9 (3.9)	
AHEI^b^	42.6 (10.4)		43.5 (9.9)		43.7 (10.2)		44.3 (10.2)		43.7 (10.0)	
Physical activity, MET-hrs/wk^b,c^	18.9 (21.4)		23.2 (31.4)		22.4 (28.7)		22.6 (31.1)		22.8 (30.4)	
Alcohol intake (gm)^b^	2.4 (4.7)		2.5 (4.8)		2.8 (5.2)		2.9 (4.5)		2.7 (4.9)	
Smoking history before pregnancy^a^										
Never		75.3		74.0		73.1		63.2		71.9
Past		18.2		19.6		20.2		26.3		20.9
Current		6.5		6.4		6.7		10.5		7.2
High blood pressure^e^		2.4		2.6		3.5		1.7		2.8
Diabetes^e^		0.3		0.2		0.2		0.5		0.3
Husbands holding a graduate degree^f^		31.9		36.5		37.1		31.1		35.9
Subjective US social/economic standing^g^	3.8 (1.3)		3.8 (1.3)		3.8 (1.4)		3.9 (1.3)		3.8 (1.5)	
Subjective community social/economic standing^g^	3.9 (1.5)		3.9 (1.6)		4.0 (1.5)		4 .0 (1.5)		3.9 (1.5)	
Household annual income category^h^	7.3 (1.7)		7.4 (1.7)		7.4 (1.7)		7.5 (1.7)		7.4 (1.7)	
US Census tract median household income^e^	62,644 (22,173)		63,142 (23,412)		62,942 (21,719)		61,272 (21,400)		62,779 (23,006)	
US Census tract % college educated^e,d^		30.6		32.1		33.2		31.6		32.4
Geographic region^I,d^										
West		20.5		14.8		12.9		10.1		13.4
Midwest		34.2		36.5		36.1		33.7		35.9
South		14.8		16.0		16.7		11.3		15.5
Northeast		30.6		32.7		34.3		44.8		35.2
Parity before first included pregnancy										
Nulliparous		20.3		21.8		22.0		20.5		21.6
One previous pregnancy		32.4		29.0		30.0		30.7		29.7
Two previous pregnancies		24.0		25.8		26.0		25.2		25.8
Three or more previous pregnancies		23.3		23.4		22.0		23.6		22.9
Mother ever^j^ depressed		19.4		21.5		22.5		27.4		22.8
Mother’s chronotype										
Definite morning type		31.9		36.0		34.4		37.4		35.6
Intermediate type		56.9		55.0		55.6		52.1		54.7
Definite evening type		11.2		9.0		10.0		10.5		9.6
Number of pregnancies, n		1683		1507		1132		491		3130
Offspring gender										
Male		46.1		45.0		48.2		49.3		46.8
Female		53.9		55.0		51.8		50.7		53.2
Offspring age at GUTS baseline 2004	11.8 (1.2)		11.6 (1.2)		11.6 (1.2)		11.6 (1.3)		11.6 (1.2)	
Offspring Tanner stage^i^	2.8 (1.3)		2.6 (1.3)		2.6 (1.3)		2.7 (1.3)		2.6 (1.3)	
Offspring weekly hours sedentary behavior^i^	4.0 (2.8)		3.9 (2.7)		3.9 (2.5)		4.1 (2.4)		3.9 (2.6)	

*AHEI* alternative healthy eating index; *METS* metabolic-equivalent hours; *SD* standard deviation

^a^Recorded on the most recent questionnaire prior to conception of first included offspring

^b^Recorded in 1991

^c^One metabolic-equivalent-hour is proportional to the amount of energy spent sitting quietly for 1 h

^d^Percentages are of non-missing values

^e^Recorded in 1989

^f^Recorded in 1999

^g^Reported in 2001, the scale had 10 levels, lower score indicates higher SES

^h^Reported in 2001, the scale had 9 levels, from 1 ≤ $15,000 to 9 ≥ $150,000

^i^At GUTS baseline 2004

^j^Self-reported physician/clinician-diagnosed depression

We did not observe an overall association between maternal night shift work before pregnancy and risk of any of depression and anxiety in their offspring (Table 2). However, there was some indication that maternal chronotype might play a role in the relationship between maternal night shift work before pregnancy and depression in offspring (Table 3). Because differences between basic and multivariable adjusted models were small, we focus on the results obtained from fully-adjusted models only (MV models 2 adjusting for the largest set of relevant covariates).

**Table 2:** Adjusted odds ratios (OR) for offspring risk of depression during childhood and adolescence *according to maternal rotating night shiftwork history before pregnancy,* using data from the Growing Up Today Study 2 from 2004 to 2013, restricted to singleton, full-term births

	History of rotating night shift work									
	Never worked rotating night shifts	< 3 years		3–5 years		≥ 6 years			Ever worked rotating night shifts	
Self-reported physician/clinician—diagnosed depression*										
Cases/participants	92/1683	99/1507		78/1132		23/491			200/3130	
	OR	OR	95% CI	OR	95% CI	OR	95% CI	P trend	OR	95% CI
Basic model^a^	1 (reference)	1.25	0.93, 1.68	1.33	0.97, 1.82	0.88	0.55, 1.40	0.63	1.22	0.94, 1.57
MV model 1^b^	1 (reference)	1.20	0.89, 1.62	1.26	0.92, 1.73	0.82	0.51, 1.33	0.82	1.17	0.90, 1.51
MV model 2^c^	1 (reference)	1.18	0.87, 1.60	1.23	0.89, 1.69	0.79	0.49, 1.27	0.99	1.14	0.88, 1.47
Self-reported physician/clinician—diagnosed depression* or SSRI** use or CESD10 ≥ 10^x^										
Cases/participants	389/1683	346/1507		263/1132		91/491			700/3130	
	OR	OR	95% CI	OR	95% CI	OR	95% CI	P trend	OR	95% CI
Basic model^a^	1 (reference)	0.99	0.84, 1.17	1.02	0.85, 1.23	0.77	0.59, 0.99	0.25	0.96	0.83, 1.11
MV model 1^b^	1 (reference)	0.98	0.83, 1.16	1.00	0.83, 1.20	0.74	0.56, 0.96	0.16	0.95	0.82, 1.10
MV model 2^c^	1 (reference)	0.97	0.82, 1.15	0.99	0.82, 1.19	0.73	0.56, 0.95	0.13	0.94	0.81, 1.09
Self-reported physician/clinician—diagnosed anxiety*										
Cases/participants	98/1683	80/1507		60/1132		25/491			165/3130	
	OR	OR	95% CI	OR	95% CI	OR	95% CI	P trend	OR	95% CI
Basic model^a^	1 (reference)	0.92	0.68, 1.25	0.94	0.68, 1.32	0.91	0.58, 1.43	0.63	0.93	0.71, 1.20
MV model 1^b^	1 (reference)	0.88	0.64, 1.20	0.90	0.64, 1.25	0.82	0.52, 1.31	0.43	0.88	0.67, 1.14
MV model 2^c^	1 (reference)	0.86	0.63, 1.18	0.86	0.61, 1.21	0.79	0.50, 1.26	0.30	0.85	0.66, 1.11
Combination of any psychiatric outcome^§^										
Cases/participants	407/1683	361/1507		272/1132		96/491			729/3130	
	OR	OR	95% CI	OR	95% CI	OR	95% CI	P trend	OR	95% CI
Basic model^a^	1 (reference)	0.99	0.84, 1.17	1.01	0.84, 1.21	0.77	0.60, 1.00	0.22	0.96	0.83, 1.11
MV model 1^b^	1 (reference)	0.97	0.82, 1.15	0.99	0.82, 1.19	0.74	0.57, 0.96	0.14	0.94	0.82, 1.09
MV model 2^c^	1 (reference)	0.97	0.82, 1.14	0.98	0.81, 1.17	0.73	0.56, 0.95	0.10	0.93	0.81, 1.08

*CESD10* Center for Epidemiologic Studies Depression Scale; *CI* confidence interval; *MV* multivariable model; *OR* odds ratio

*Assessed in 2013 (timing: before 2005; between 2006 and 2008; between 2009 and 2011, 2012 +)

**Assessed in 2013 (intake in the past 12 months)

^x^Assessed in 2011 and 2013

^§^Defined as self-reported physician/clinician—diagnosed depression ^*^ OR SSRI ** use OR CESD10 ≥ 10 OR self-reported physician/clinician—diagnosed anxiety

^a^Adjusted for offspring gender (boy/girl) and offspring age at GUTS baseline 2004

^b^Additionally adjusted for maternal age at pregnancy, smoking status before pregnancy (never, current, past), alternative healthy eating score (quintiles), physical activity (METs hours/week; quintiles), husband’s education (less than 2 year college, 4 year college, grad school), parity (nulliparity, 1, 2, 3 + previous pregnancies), BMI before pregnancy (< 25, 25–29, ≥ 30 kg/m^2^), geographic region (West, Midwest (reference), South, Northeast) and Census tract education rate in 1989

^c^Additionally adjusted for maternal depression diagnosis (yes/no)

**Table 3:** Adjusted odds ratios (OR) for offspring risk of depression during childhood and adolescence *according to maternal rotating night shiftwork history before pregnancy,* using data from the Growing Up Today Study 2 from 2004 to 2013, restricted to singleton, full-term births, stratified by maternal chronotype (Definite morning type vs. NOT definite morning type)

	History of rotating night shift work									
	Never worked rotating night shifts	< 3 years		3–5 years		≥ 6 years			Ever worked rotating night shifts	
Self-reported physician/clinician—diagnosed depression*										
Definite morning types										
Cases/participants	20/542	35/528	35/528		30/391		13/182			78/1101
	OR	OR	95% CI	OR	95% CI	OR	95% CI	P trend	OR	95% CI
Basic model^a^	1 (reference)	1.90	1.08, 3.36	2.23	1.24, 3.99	2.07	1.01, 4.23	0.01	2.04	1.24, 3.38
MV model 1^b^	1 (reference)	1.85	1.04, 3.30	2.20	1.22, 3.97	2.03	0.95, 4.36	0.02	2.00	1.21, 3.33
MV model 2^c^	1 (reference)	1.81	1.02, 3.23	2.16	1.19, 3.89	1.93	0.91, 4.12	0.03	1.95	1.17, 3.24
Intermediate and definite evening types										
Cases/participants	70/1138	64/973		48/740		10/309			122/2022	
	OR	OR	95% CI	OR	95% CI	OR	95% CI	P trend	OR	95% CI
Basic model^a^	1 (reference)	1.10	0.77, 1.57	1.11	0.75, 1.63	0.52	0.27, 1.03	0.34	1.01	0.74, 1.37
MV model 1^b^	1 (reference)	1.05	0.73, 1.51	1.04	0.70, 1.54	0.48	0.24, 0.94	0.20	0.96	0.70, 1.31
MV model 2^c^	1 (reference)	1.03	0.72, 1.49	1.00	0.68, 1.49	0.45	0.23, 0.90	0.13	0.93	0.68, 1.28
							(P Interaction) = 0.03			
Self-reported physician/clinician—diagnosed depression* or SSRI** use or CESD10 ≥ 10^x^										
Definite morning types										
Cases/participants	121/542	125/528		91/391		35/182			251/1101	
	OR	OR	95% CI	OR	95% CI	OR	95% CI	P trend	OR	95% CI
Basic model^a^	1 (reference)	1.07	0.81, 1.43	1.06	0.77, 1.46	0.83	0.54, 1.28	0.66	1.03	0.80, 1.32
MV model 1^b^	1 (reference)	1.06	0.79, 1.42	1.08	0.78, 1.49	0.81	0.52, 1.25	0.68	1.02	0.79, 1.32
MV model 2^c^	1 (reference)	1.05	0.78, 1.41	1.07	0.78, 1.49	0.80	0.52, 1.23	0.65	1.02	0.79, 1.32
Intermediate and definite evening types										
Cases/participants	266/1138	219/973		172/740		56/309			447/2022	
	OR	OR	95% CI	OR	95% CI	OR	95% CI	P trend	OR	95% CI
Basic model^a^	1 (reference)	0.95	0.77, 1.17	1.01	0.81, 1.26	0.74	0.53, 1.02	0.30	0.94	0.79, 1.12
MV model 1^b^	1 (reference)	0.95	0.77, 1.17	0.98	0.78, 1.24	0.71	0.51, 0.99	0.20	0.93	0.77, 1.11
MV model 2^c^	1 (reference)	0.94	0.77, 1.16	0.97	0.77, 1.22	0.70	0.50, 0.97	0.15	0.91	0.76, 1.09
							P (Interaction) = 0.96			

*CESD10* center for epidemiologic studies depression scale; *CI* confidence interval; *MV* multivariable model; *OR* odds ratio

*Assessed in 2013 (timing: before 2005; between 2006 and 2008; between 2009 and 2011, 2012 +)

**Assessed in 2013 (intake in the past 12 months)

^x^Assessed in 2011 and 2013

^a^Adjusted for offspring gender (boy/girl) and offspring age at GUTS baseline 2004

^b^Additionally adjusted for maternal age at pregnancy, smoking status before pregnancy (never, current, past), alternative healthy eating score (quintiles), physical activity (METs hours/week; quintiles), husband’s education (less than 2 year college, 4 year college, grad school), parity (nulliparity, 1, 2, 3 + previous pregnancies), BMI before pregnancy (< 25, 25–29, ≥ 30 kg/m^2^), geographic region (West, Midwest (reference), South, Northeast) and Census tract education rate in 1989

^c^Additionally adjusted for maternal depression diagnosis (yes/no)

We found no association between maternal night shift work and offspring depression, regardless of the case definition that was applied: e.g. risk of physician/clinician-diagnosed depression (OR_ever nightwork _= 1.14; 95% CI, 0.88, 1.47); risk of depression using the broader case definition (physician-diagnosed depression or SSRI use or CESD10≥ 10); OR_ever nightwork _= 0.94; 95% CI, 0.81, 1.09) (Table 2). Also, children born to mothers who had worked night shifts had a similar risk of physician-diagnosed anxiety (OR_ever nightwork _= 0.85; 95% CI, 0.66, 1.11) and similar risk of any outcome (OR_ever nightwork _= 0.93; 95% CI, 0.81, 1.08), compared to children born to mothers without shiftwork history. Similarly, duration of night shift work history was not associated with risk of any of the outcomes considered (all P_Trend _> 0.10) (Table 2).

We conducted analyses stratified by maternal chronotype (Table 3). Compared to offspring of women without a history of rotating night shift work, offspring of women with any rotating night work before pregnancy had a significantly elevated risk of physician-diagnosed depression among women who were definite morning chronotypes (OR_ever nightwork _= 1.95; 95% CI, 1.17, 3.24), whereas this was not the case for offspring of women with intermediate or evening chronotypes (OR_ever nightwork _= 0.93; 95% CI, 0.68, 1.28; P_Interaction _= 0.03). Similar indications of effect modification were not observed for the alternative—Boolean OR—case definition of depression (based on reporting a physician/clinician-diagnosed depression, regular antidepressant use, or the presence of clinically significant depressive symptom) or the other considered outcomes (P_interaction_ for broader depression definition = 0.96, for self-reported anxiety = 0.24, for any psychological outcome = 0.96).

### Maternal night shift work exposure during pregnancy

A detailed description of maternal and offspring characteristics for the subsample with available exposure information during pregnancy can be found elsewhere [47].

We restricted our analyses to the broad endpoints of any depression or anxiety outcome, as well as the depression definition based on physician-diagnosed depression, SSRI use, or depressive symptoms (CESD-10 ≥ 10). Again, results from basic and multivariable models did not differ markedly (Supplemental Table 1), so we focus on results from the fully-adjusted models (MV model 2) only. Briefly, risks of depression and anxiety for children of women with or without night shift work exposure during pregnancy were not significantly different (risk of depression OR_ever nightwork _= 1.14; 95% CI, 0.68, 1.94; risk of any psychiatric outcome OR_ever nightwork_ = 1.17; 95% CI, 0.70, 1.98).

## Discussion

In this study including mothers enrolled in NHSII and their offspring participating in GUTS2, we found no overall associations of maternal history of rotating night shift work before pregnancy or shift work exposure during pregnancy with depression or anxiety in their children. Among mothers who were definite morning chronotypes, there was significantly higher risk of physician/clinician-diagnosed depression offspring of mothers who participated in rotating night work before pregnancy compared to offspring of mothers who did not. Hence, there is some indication that biologically-based maternal preferences in sleep timing might play a role in the relationship between maternal night shift work before pregnancy and depression outcomes, suggesting that social and biological stress induced by an extreme mismatch of preferred and actual sleeping times might have an impact on offspring mental health. Interestingly, we identified this effect modification only for self-reported physician/clinician-diagnosed depression and not for any of the other considered outcomes, including a broader depression definition that also included antidepressant use and depressive symptoms. Possible explanations are that self-reported physician/clinician-diagnosed depression may capture more severe depression cases, may have higher specificity and precision than the broad definition, or may indicate earlier age-at-onset cases.

Rotating night shift work, an established social and biological stressor [34, 48], and chronotype have independently been linked to increased risk of depression [11, 12, 14, 45, 49, 50], and an interaction of chronotype and work hours has been reported previously for chronic disease outcomes [19, 20] within the same person. Growing literature, including animal and human studies, supports the hypothesis that prenatal programming can impact psychiatric disorders, which may be driven by alterations of the HPA axis and, in turn, effects on the circadian and limbic system [10]. Nevertheless, to the best of our knowledge, there is no study so far that investigated the intergenerational associations of maternal night shift work exposure before and during pregnancy to offspring mental health outcomes.

We consider the longitudinal follow-up of mothers and children, together with detailed information on determinants of maternal lifestyle and social economic status, to be unique strengths of our study.

Limitations should also be mentioned. The timing of the supplemental pregnancy questionnaire led to a small time window of overlap between mothers with available exposure information during pregnancy and children enrolled in GUTS2. Therefore, we had limited power to explore relationships between pregnancy shift work exposure and mental health outcomes in detail.

Further, the self-reported nature of our outcome definition could have contributed to misclassification. While the use of the CESD-10 scale has been validated as a screening tool [37] and the validity of self-reported clinician/physician diagnosed depression has been considered adequate in adults [51], mental health disorders might be under-diagnosed in the general population and especially in the adolescent and young adults [52]. Nevertheless, outcome misclassification was likely non-differential with respect to shift work exposures and maternal chronotype.

Similarly, maternal night shift work exposure is based on self-reports and crude assessments, asking about the duration nurses had worked at least 3 nights per month in addition to working days or evenings. Hence, the exposure may have been misclassified. Most likely the potential misclassification occurred at random and therefore could have biased our results only towards the null.

Also, chronotype was assessed by one sub-item of the Morningness-Eveningness Questionnaire [42] and rather distant in time from the exposure assessment. However, it has been shown that this single-item measure correlates well with the overall Morningness-Eveningness score [53] and that, even though chronotype can change with age, these changes are slow in pace once adulthood is reached [54] and tend to affect everyone equally (i.e., evening chronotypes will always remain more evening types relative to others, even when factoring in the effects of age).

In conclusion, there was no overall association between duration of maternal night shift work before or during pregnancy and offspring depression or anxiety in our study. However, results from stratified analyses indicate that maternal chronotype might play a role in the relationship between maternal night shift work before pregnancy and depression outcomes in their offspring. Future studies with more refined exposure assessments and more detailed longitudinal measures of psychiatric outcomes throughout childhood, adolescence, and adulthood are needed to explore these relationships in more detail.

## Electronic supplementary material

Below is the link to the electronic supplementary material.

Supplementary material 1 (DOCX 20 kb)
