# Supplementary material for: Night shift work before and during pregnancy in relation to depression and anxiety in adolescent and young adult offspring
Source: Eur J Epidemiol. 2019 May 13;34(7):625–35. doi: 10.1007/s10654-019-00525-2 (PMC6548754; doi:10.1007/s10654-019-00525-2)
Supplement: Supplementary file 1 — Supplementary material 1 (DOCX 20 kb) [file 10654_2019_525_MOESM1_ESM.docx]

**Supplemental Table 1. Maternal and Offspring Characteristics by Rotating Night Shift Work History Before Pregnancy, for 4,044 Mothers of a Total of 4,813 Children, Born Between 1989 and 1995, Enrolled in the Growing Up Today Study 2**

| **Characteristic** | **Average number of night shifts/month during pregnancy** | | | |
| --- | --- | --- | --- | --- |
|  | **None** | | **Any no. nights/mo** | |
|  | **(n=**435**)** | | **(n=110)** | |
|  | Mean (SD) | % ^d^ | Mean (SD) | % |
| Maternal age at delivery | 34.9 (4.5) |  | 34.0 (2.8) |  |
| BMI before pregnancy,^a^ | 23.9 (4.5) |  | 23.5 (3.8) |  |
| AHEI,^b^ | 42.7 (9.9) |  | 41.0 (10.5) |  |
| Physical activity, MET-hrs /wk, ^b^,^c^ | 23.2 (34.0) |  | 19.3 (22.2) |  |
| Alcohol intake (gm) ^b^ | 2.3 (4.0) |  | 2.2 (3.2) |  |
| Smoking history before pregnancy,^a^ |  |  |  |  |
| Never |  | 76.0 |  | 72.7 |
| Past |  | 18.7 |  | 21.8 |
| Current |  | 5.3 |  | 5.5 |
|  |  |  |  |  |
| High blood pressure ^e^ |  | 2.5 |  | 0.0 |
| Diabetes ^e^ |  | 0.0 |  | 0.0 |
| Husbands holding a graduate degree ^f,^ |  | 30.8 |  | 26.4 |
| Subjective US social/economic standing ^g^ | 3.8 (1.3) |  | 4.0 (1.2) |  |
| Subjective community social/economic standing ^g^ | 4.0 (1.5) |  | 4.0 (1.6) |  |
| Household annual income category ^h^ | 7.3 (1.6) |  | 7.2 (1.7) |  |
| US Census tract median household income ^e^ | 62,511 (23,179) |  | 57,487 (17,893) |  |
| US Census tract % college educated ^e,d^ |  | 31.7 |  | 27.0 |
| Geographic region ^I,d^ |  |  |  |  |
| West |  | 14.4 |  | 16.5 |
| Midwest |  | 40.8 |  | 41.3 |
| South |  | 11.8 |  | 13.8 |
| Northeast |  | 33.0 |  | 28.4 |
| Parity before first included pregnancy |  |  |  |  |
| Nulliparous |  | 6.0 |  | 8.5 |
| One previous pregnancy |  | 28.3 |  | 29.7 |
| Two previous pregnancies |  | 32.7 |  | 30.9 |
| Three or more previous pregnancies |  | 33.0 |  | 30.9 |
| History of rotating night shift work, % |  |  |  |  |
| None |  | 34.7 |  | 20.00 |
| <3 years |  | 36.6 |  | 32.7 |
| 3 – 5 years |  | 21.6 |  | 30.0 |
| ≥ 6 years |  | 7.1 |  | 17.3 |
| Mother ever ^j^ depressed |  | 20.2 |  | 20.0 |
| Mother’s chronotype |  |  |  |  |
| Definite morning type |  | 29.6 |  | 27.6 |
| Intermediate type |  | 60.6 |  | 51.0 |
| Definite evening type |  | 9.8 |  | 21.4 |
| **Number of pregnancies, n** |  | 435 |  | 110 |
| Offspring gender |  |  |  |  |
| Male |  | 45.3 |  | 47.3 |
| Female |  | 54.7 |  | 52.7 |
| Offspring age at GUTS baseline 2004 | 10.0 (0.6) |  | 10.0 (0.6) |  |
| Offspring Tanner stage ^i^ | 3.5 (2.3) |  | 3.7 (2.1) |  |
| Offspring weekly hrs sedentary behavior ^i^ | 3.5 (2.3) |  | 3.7 (2.1) |  |

Abbreviations: AHEI, alternative healthy eating index; METS, metabolic-equivalent hours; SD, standard deviation; yrs. years

^a^ Recorded on the most recent questionnaire prior to conception of first included offspring

^b^ Recorded in 1991

^c^ One metabolic-equivalent-hour is proportional to the amount of energy spent sitting quietly for one hour.

^d^ Percentages are of non-missing values.

^e^ Recorded in 1989

^f^ Recorded in 1999

^g^ Reported in 2001, the scale had 10 levels, lower score indicates higher SES

^h^ Reported in 2001, the scale had 9 levels, from 1= <$15,000 to 9= ≥$150,000

^i^ At GUTS baseline 2004)

^j^ Self-reported physician/clinician-diagnosed depression

**Supplemental Table 2. Adjusted odds ratios (OR) for offspring risk of depression or any psychiatric outcome during childhood and adolescence by night shift exposure during pregnancy using data from the Growing Up Today Study 2 from 2004 to 2013, restricted to singleton, full-term births.**

|  | **Average number of night shifts/month during pregnancy** |  | |  | |
| --- | --- | --- | --- | --- | --- |
|  | **None** | **Any no. nights/mo** | | | |
| **Self-reported physician/clinician - diagnosed depression^*^ OR SSRI^**^ use OR CESD10≥10 ^x^** | | |  | |  |
| Cases/participants | 93/435 | 25/110 | | | |
|  | **OR** | **OR** | | **95% CI** | |
| Basic model ^a^ | 1 (reference) | 1.09 | | 0.66, 1.80 | |
| MV model 2 ^b^ | 1 (reference) | 1.14 | | 0.68, 1.93 | |
| MV model 3 ^c^ | 1 (reference) | 1.14 | | 0.68, 1.94 | |
| **Combination of any psychiatric outcome^§^** | | | |  | |
| Cases/participants | 98/435 | 26/110 | | | |
|  | **OR** | **OR** | | **95% CI** | |
| Basic model ^a^ | 1 (reference) | 1.07 | | 0.65, 1.76 | |
| MV model 2 ^b^ | 1 (reference) | 1.17 | | 0.70, 1.98 | |
| MV model 3 ^c^ | 1 (reference) | 1.17 | | 0.70, 1.98 | |

* Assessed in 2013 (timing: before 2005; between 2006 and 2008; between 2009 and 2011, 2012 +)

** Assessed in 2013 (intake in the past 12 months)

^x^ Assessed in 2011 and 2013

^§^ Defined as self-reported physician/clinician - diagnosed depression^*^ or SSRI^**^ use or CESD10≥10 or self-reported physician/clinician – diagnosed anxiety

Abbreviations: CESD10, Center for Epidemiologic Studies Depression Scale; CI, confidence interval; MV, multivariable model; OR, odds ratio

^a^ Adjusted for offspring gender (boy/girl)

**^b^** Additionally adjusted for maternal age at pregnancy, smoking status before pregnancy (never, current, past), alternative healthy eating score (quintiles), physical activity (METs hours/week; quintiles), husband’s education (less than 2yr college, 4yr college, grad school), parity (nulliparity, 1, 2, 3+ previous pregnancies), BMI before pregnancy (<25, 25-29, ≥30 kg/m^2^), geographic region ( west, midwest (reference), south, northeast) and Census tract education rate in 1989, history of rotating night shift work (never, <3 yrs, 3-5 yrs, ≥ 6 yrs)

^c^ Additionally adjusted for maternal depression diagnosis (yes/no)
